# Confounding and Statistical Significance of Indirect Effects: Childhood Adversity, Education, Smoking, and Anxious and Depressive Symptomatology

**DOI:** 10.3389/fpsyg.2017.01317

**Published:** 2017-08-02

**Authors:** Mashhood Ahmed Sheikh

**Affiliations:** Health Services Research Unit, Department of Community Medicine, University of Tromsø Tromsø, Norway

**Keywords:** direct effect, proportion of mediated effect, decomposition, mediation, confounding, anxiety, depression, longitudinal

## Abstract

The life course perspective, the risky families model, and stress-and-coping models provide the rationale for assessing the role of smoking as a mediator in the association between childhood adversity and anxious and depressive symptomatology (ADS) in adulthood. However, no previous study has assessed the independent mediating role of smoking in the association between childhood adversity and ADS in adulthood. Moreover, the importance of mediator-response confounding variables has rarely been demonstrated empirically in social and psychiatric epidemiology. The aim of this paper was to (i) assess the mediating role of smoking in adulthood in the association between childhood adversity and ADS in adulthood, and (ii) assess the change in estimates due to different mediator-response confounding factors (education, alcohol intake, and social support). The present analysis used data collected from 1994 to 2008 within the framework of the Tromsø Study (*N* = 4,530), a representative prospective cohort study of men and women. Seven childhood adversities (low mother's education, low father's education, low financial conditions, exposure to passive smoke, psychological abuse, physical abuse, and substance abuse distress) were used to create a childhood adversity score. Smoking status was measured at a mean age of 54.7 years (Tromsø IV), and ADS in adulthood was measured at a mean age of 61.7 years (Tromsø V). Mediation analysis was used to assess the indirect effect and the proportion of mediated effect (%) of childhood adversity on ADS in adulthood via smoking in adulthood. The test-retest reliability of smoking was good (Kappa: 0.67, 95% CI: 0.63; 0.71) in this sample. Childhood adversity was associated with a 10% increased risk of smoking in adulthood (Relative risk: 1.10, 95% CI: 1.03; 1.18), and both childhood adversity and smoking in adulthood were associated with greater levels of ADS in adulthood (*p* < 0.001). Smoking in adulthood did not significantly mediate the association between childhood adversity and ADS in adulthood. However, when education was excluded as a mediator-response confounding variable, the indirect effect of childhood adversity on ADS in adulthood was statistically significant (*p* < 0.05). This study shows that a careful inclusion of potential confounding variables is important when assessing mediation.

## Introduction

In social and psychiatric epidemiology, one of the most studied associations is that between childhood adversity, smoking, and health-related outcomes in adulthood (Patten et al., 2016; Sheikh et al., 2016a; Tani et al., 2016; Sheikh, 2017). Previous studies have shown that childhood adversity increases the risk of certain adult behaviors that can increase the risk of anxiety and depression (Sheikh et al., 2016a; Tani et al., 2016). In particular, smoking has often been associated with childhood adversity (Power and Hertzman, 1997; Anda et al., 1999; Gilman et al., 2003; Kestilä et al., 2006; Sheikh et al., 2016a) and with anxiety and depression in later life (Fluharty et al., 2016; Le et al., 2016; Sheikh et al., 2016a).

Chronic stress (the cumulative load of day-to-day stresses) in childhood caused by socioeconomic and psychosocial adversity may foster impaired self-regulation, which in turn may affect unhealthy lifestyle choices such as smoking (McEwen, 2000; Mulvihill, 2005; Miller et al., 2011; Le et al., 2016; Sheikh, 2017). Previous psychological research has shown that childhood adversity is associated with higher impulsivity in adolescence (Lau et al., 1999; Robbins and Bryan, 2004; Miller et al., 2011). In turn, adolescents with higher impulsivity are more likely to smoke (Robbins and Bryan, 2004; Balevich et al., 2013; Mitchell and Potenza, 2014; Stautz et al., 2016). Other studies have shown that childhood adversity is associated with smoking in adulthood independent of DSM-IV psychopathology (Spratt et al., 2009; Fuller-Thomson et al., 2013). Smoking may serve as self-medication, as a coping strategy to induce a relaxed state (Warburton, 1987; Mulvihill, 2005), or as a method of compensating for deficiencies in social and emotional development (Repetti et al., 2002; Gould and Martindale, 2007; Strine et al., 2012; Prock, 2015). Social and emotional impairments, and deficits in interpersonal development and affect regulation, have also been proposed as consequences of childhood adversity (Kim and Cicchetti, 2010; Moretti and Craig, 2013; Dvir et al., 2014; Stikkelbroek et al., 2016). The life course perspective (Elder, 1985), the risky families model (Repetti et al., 2002), and stress-and-coping models (Pearlin et al., 2005) provide the rationale for assessing the role of smoking as a mediator in the association between childhood adversity and anxious and depressive symptomatology (ADS) in adulthood.

Analytically, if the magnitude of the association between smoking and ADS in adulthood remains strong after controlling for childhood adversity and confounding variables, and the direct effect of childhood adversity (adjusted for smoking and confounding variables) on ADS in adulthood remains strong, it indicates that both the initial stressor (childhood adversity) and the secondary stressor (smoking) exert a unique negative effect on ADS in adulthood. However, if the magnitude of the association between childhood adversity and ADS in adulthood after controlling for smoking and confounding variables is close to null, and the association between smoking and ADS remains strong (after controlling for childhood adversity and confounding variables), it indicates that smoking influences ADS in place of childhood adversity (i.e., almost all of the influence of childhood adversity on ADS in adulthood is mediated by smoking). However, the causal interpretation of these inferences are subject to a number of assumptions (Robins and Greenland, 1992; Cole and Hernán, 2002; Sheikh et al., 2016a,b; Sheikh, 2017).

The association between childhood adversity and ADS in adulthood is likely confounded by genetic dispositions (Sheikh, 2017). Indeed, almost 40–70% (depending on the stringency of the definition) of one's risk of anxiety and depression is genetically determined (Kendler et al., 1993a; McGuffin et al., 1996; Heim and Binder, 2012). Parental history of mental and physical chronic conditions may serve as a crude proxy for genetic dispositions (Sheikh, 2017). Experts in mediation analysis have repeatedly pointed out that an unbiased decomposition of total effects into direct and indirect effects relies on a number of assumptions, including no unmeasured (or unaccounted for) mediator-response confounding variables (Robins and Greenland, 1992; Cole and Hernán, 2002). Several studies have shown that smoking is closely associated with education, alcohol intake, and social support (Donovan and Jessor, 1985; Shiffman et al., 1994; Ary et al., 1999; Haustein, 2006; Gilman et al., 2008; Jackson et al., 2010). Other reports suggest that these factors are likely associated with childhood adversity and ADS later in life (Kauhanen et al., 2011; Lemos et al., 2012; Kim et al., 2013; Mersky et al., 2013; Campbell et al., 2016; Nurius et al., 2016). Consequently, several studies have indicated that education, alcohol intake, and social support mediate the association between childhood adversity and ADS in adulthood (Nurius et al., 2015; Openshaw et al., 2015; Shevlin et al., 2015; Lê-Scherban et al., 2016; Muller, 2016; Ni et al., 2016; Sheikh et al., 2016a,b; Tani et al., 2016; Korhonen et al., 2017; Kwon and Park, 2017; Lee et al., 2017; Markkula et al., 2017; Wielaard et al., 2017) and suggested that these are important mediator-response confounding factors that must be included in analytical models in order to assess the indirect effect of childhood adversity on ADS in adulthood via smoking. It is plausible that if the mediating role of smoking is assessed without controlling for key mediator-response confounding variables, the observed indirect effect may be biased upwards (or will be statistically significant), not because smoking alone mediates the association between childhood adversity and ADS, but because of the correlation between smoking and an unmeasured (or unaccounted for) mediator-response confounder. Therefore, controlling for potential mediator-response confounding factors (e.g., education, alcohol intake, and social support) is crucial in order to infer that smoking “alone” mediates the association between childhood adversity and ADS in adulthood.

Often a mediator-response confounder goes unmeasured; when this happens one basically has to invent a scenario to assess how an unmeasured mediator-response confounder would affect the direct and indirect effects. Indeed, the sensitivity analysis that has been proposed for this (VanderWeele and Arah, 2011; VanderWeele, 2015) consists of inventing an unmeasured mediator-response confounder, as well as its “unobserved” association with the exposure, mediator, and response. This invented information is then used to assess how much the direct and indirect effects would change if that mediator-response confounder was measured. This process of using invented information is unlikely to have any practical significance, unless empirical studies can show convincing evidence that accounting for such a mediator-response confounder significantly affects the direct and indirect effects.

The aim of the present study was (i) to assess the mediating role of smoking in the association between childhood adversity and ADS in adulthood, and (ii) to assess the change in estimates due to different mediator-response confounding factors (education, alcohol intake, and social support).

## Methods

### Study population

The Tromsø Study is a longitudinal prospective cohort study and its participants are considered representative of the adult population residing in the municipality of Tromsø (Jacobsen et al., 2012). Between 1974 and 2008, six waves of the Tromsø Study were conducted (referred to as Tromsø I-VI) (Jacobsen et al., 2012). The present analysis includes data collected from 1994 to 2008.

To be eligible for the present analyses, participants had to have attended all of the following waves: Tromsø IV (1994–1995), Tromsø V (2001–2002), and Tromsø VI (2007–2008) (*N* = 4,530). The study sample included participants aged 25–74 years at Tromsø IV, 32–81 years at Tromsø V, and 38–87 years at Tromsø VI.

### Ethical approval

This investigation was carried out in accordance with the latest version of the Declaration of Helsinki. The Tromsø Study has been approved by the Regional Committee for Medical and Health Research Ethics, the Data Inspectorate, and the Norwegian Directorate of Health. Written informed consent was obtained from all individual participants included in the study.

### Study variables

#### Exposure (childhood adversity)

The present analysis used seven indicators of childhood adversity (low mother's education, low father's education, low childhood financial conditions, three kinds of adverse childhood experiences, and exposure to passive smoke in childhood) measured retrospectively in Tromsø IV and Tromsø VI, to create a childhood adversity score. Participants received 1 point for each adversity that was present in their childhood, thus scores ranged from 0 to 7. Independent associations between each childhood adversity and ADS has been shown in earlier studies that also used data from the Tromsø Study (Sheikh et al., 2014, 2016a,b).

Parental education was used as an indicator of social background. Mother's and father's education was obtained separately on a 5-level scale in Tromsø VI as: (1) college or university (4 years or more); (2) college or university (less than 4 years); (3) high school diploma; (4) vocational school or technical school; and (5) primary and secondary school or similar (i.e., 7–10 years of schooling). The lowest parental education level, i.e., parental primary and secondary school or similar, was considered a childhood adversity. Childhood financial conditions was used as the indicator of economic background, and was obtained in Tromsø IV as: “How was your family's financial situation when you were a child?” Participants replied using a 4-point scale ranging from *very difficult* (1) to *very good* (4). Those who answered difficult or very difficult were considered to have this childhood adversity. The test-retest reliability of childhood financial conditions was good (Kappa_weighted_: 0.61, 95% confidence interval [CI] 0.59; 0.63) in Tromsø study (Sheikh et al., 2016b; Sheikh, 2017). Information on adverse childhood experiences were obtained in Tromsø VI as: “Have you over a long period experienced any of the following? (as a child),” followed by three types of traumatic experiences: (i) being tormented, or threatened with violence; (ii) being beaten, kicked, or the victim of other types of violence; and (iii) someone in your close family using alcohol or drugs in such a way that caused you worry. Each of these adverse childhood experiences was considered a childhood adversity. The internal reliability of these adverse childhood experiences was good in the Tromsø Study (Sheikh, 2017). Exposure to passive smoke in childhood was obtained in Tromsø IV as: “Did any of the adults smoke at home while you were growing up?” Those reporting “yes” were considered to have this childhood adversity. The test-retest reliability of exposure to passive smoke in childhood was good (Kappa: 0.76, 95% CI: 0.74; 0.78) in this sample (Sheikh, 2017). A composite variable was constructed as sum of the seven childhood adversities (mean: 2.91, SD: 1.11).

#### Mediator (daily smoking)

Daily smoking was measured in Tromsø IV (mean age of participants: 54.7 years) as: “Do you smoke cigarettes daily?” (yes = 1/no = 0). Although the smoking status of the participants may change during follow-up, the test-retest reliability was good (Kappa: 0.67, 95% CI: 0.63; 0.71) in this sample.

#### Response (anxious and depressive symptomatology)

Anxious and depressive symptomatology was measured in the Tromsø V (mean age of participants: 61.7 years) using the Hopkins Symptom Checklist (HSCL-10). HSCL-10 consists of 10 items on a four-point scale, ranging from *not at all* (1) to *extremely* (4). The HSCL-10 had an acceptable degree of internal consistency in this sample (Cronbach's alpha: 0.86, mean inter-item correlation: 0.42, McDonald's omega coefficient for composite reliability: 0.87). In accordance with Strand et al. (2003), an HSCL-10 score between 1 and 4 was assigned by dividing the total score (sum of the 10 indicators) by the total number of items, where 4 represented the highest and 1 represented the lowest score for ADS (mean: 1.25, standard deviation [SD]: 0.33). An HSCL-10 score of 1.85 has been proposed as a cut-to predict diagnosed anxiety and depression (Strand et al., 2003; Kvamme et al., 2011; Sheikh et al., 2016a). Earlier studies using data from the Tromsø Study have suggested that over 9.3% of the adult population (age 30–89 years) in Tromsø could be classified as clinically significant cases of ADS with this cut-off (Sheikh et al., 2016a). Based on information from clinical interviews, previous literature has indicated that 50–60% of cases detected by the HSCL-10 could actually be categorized as having clinically significant anxiety and depression (Strand et al., 2003; Kvamme et al., 2011).

#### Confounding variables

The potential confounding variables age, gender, living in Norway at age 1 year, history of psychological problems in the mother/father, parental history of heart attack, angina pectoris, cerebral stroke/brain hemorrhage, osteoporosis, stomach/duodenal ulcer, asthma, diabetes, and dementia were chosen based on *a priori* knowledge of the association between childhood adversity, smoking and ADS (Wender et al., 1986; Kaslow et al., 1994; Kaplow and Widom, 2007; Pollak, 2008; Bet et al., 2009; Miller et al., 2011; Melas et al., 2013; Agerup et al., 2015; Plant et al., 2015; South et al., 2015; Fandiño-Losada et al., 2016; Sheikh, 2017). Valid information on age and gender was obtained from Statistics Norway by using the unique personal identification number of each participant. The test-retest reliability of history of psychological problems of the mother and father in Tromsø Study were Kappa: 0.57 (95% CI: 0.52; 0.62) and Kappa: 0.61 (95% CI: 0.53; 0.69), respectively (Sheikh, 2017).

#### Mediator-response confounding factors

Previous research suggests that education, alcohol intake, and social support are potential smoking-ADS confounding factors (Lahelma et al., 2006; Comijs et al., 2007; Eugenia Alvarado et al., 2007; Vranceanu et al., 2007; Mossakowski, 2008; Nicholson et al., 2008; Banou et al., 2009; Powers et al., 2009; Hill et al., 2010; Korkeila et al., 2010; McKenzie et al., 2010, 2011; Ford et al., 2011; Salazar et al., 2011). Participant's education was obtained on the same 5-level scale used for parental education (mean: 3.87, SD: 1.35). Although the education level of the participants may increase during 13 years of follow-up, the test-retest reliability was very good (Kappa: 0.91, 95% CI: 0.91; 0.92) in this sample. Alcohol intake was measured with four indicators: alcohol frequency (times/month), beer frequency (times/fortnight), wine frequency (times/fortnight), and spirit frequency (times/fortnight). Social support was measured with two indicators: number of friends and perceived social support.

### Statistical analysis

All statistical analyses were conducted using Stata version 14. Kappa agreement was used to assess the test-retest reliability of variables that were measured in at least two waves of the Tromsø Study. The kappa coefficients summarize the total agreement beyond that expected by chance (Sheikh et al., 2016c). Ninety-five percent confidence intervals (CIs) for kappa statistic were estimated with the analytical method (Fleiss et al., 2003) in Stata (Reichenheim, 2004). Established benchmarks (Sheikh et al., 2016c) for rating the strength of kappa agreements (poor: <0.20; fair: >0.20 to ≤0.40; moderate: >0.40 to ≤0.60; good: >0.60 to ≤0.80; and very good: >0.80 to ≤1.00) were used.

Missing values were generated with multiple imputation (with chained equations) to avoid any bias in the association of interest introduced by excluding individuals with missing data. One hundred multiple datasets were generated to help account for the uncertainty in the imputation procedure. In order to increase the predictive power of the imputation procedure, all indicators of the HSCL-10 were included in the imputation models. A comparison between the complete-case (excluding missing) and the imputed dataset is presented with proportions (%), and mean (standard error, SE) (Table 1). Both the unadjusted (crude) and adjusted estimates are presented.

**Table 1:** General characteristics of the study sample (*n* = 4,530).

**Characteristics**		**Complete-case dataset**	**Imputed data**
		***n* (%)**	**(%)**
**EXPOSURE-MEDIATOR, MEDIATOR-RESPONSE, AND EXPOSURE-RESPONSE CONFOUNDING VARIABLES**			
Age (in 1994)	Mean (SE)	54.69 (0.15)	−^b^
	25–34	302 (6.7)	−^b^
	35–44	352 (7.8)	−^b^
	45–54	1,327 (29.3)	−^b^
	55–64	1,852 (40.8)	−^b^
	65–74	697 (15.4)	−^b^
Gender	Male	1,849 (40.8)	−^b^
	Female	2,681 (59.2)	−^b^
Living in Norway at age 1 year^a^	Yes	4,081 (98.4)	98.4
	No	66 (1.6)	1.6
History of psychological problems, mother	Yes	261 (5.8)	−^b^
	No	4,269 (94.2)	−^b^
History of psychological problems, father	Yes	114 (2.5)	−^b^
	No	4,416 (97.5)	−^b^
Parental history of heart attack	Yes	566 (12.5)	−^b^
	No	3,964 (87.5)	−^b^
Parental history of angina pectoris	Yes	978 (21.6)	−^b^
	No	3,552 (78.4)	−^b^
Parental history of cerebral stroke/brain hemorrhage	Yes	1,043 (23.0)	−^b^
	No	3,487 (77.0)	−^b^
Parental history of osteoporosis	Yes	330 (7.3)	−^b^
	No	4,200 (92.7)	−^b^
Parental history of stomach or duodenal ulcer	Yes	559 (12.3)	−^b^
	No	3,971 (87.7)	−^b^
Parental history of asthma	Yes	455 (10.0)	−^b^
	No	4,075 (90.0)	−^b^
Parental history of diabetes	Yes	6[-0.1pt]71 (14.8)	−^b^
	No	3,859 (85.2)	−^b^
Parental history of dementia	Yes	459 (10.1)	−^b^
	No	4,071 (89.9)	−^b^
**MEDIATOR-RESPONSE CONFOUNDING VARIABLES**			
Education^a^^,^ ^c^	Mean (SE)	3.87 (0.02)	3.87 (0.02)
Alcohol frequency (times/month)^a^	Mean (SE)	3.00 (0.07)	2.90 (0.06)
Beer frequency (glasses/fortnight)^a^	Mean (SE)	1.21 (0.04)	1.14 (0.04)
Wine frequency (glasses/fortnight)^a^	Mean (SE)	1.58 (0.05)	1.52 (0.04)
Spirit frequency (glasses/fornight)^a^	Mean (SE)	1.31 (0.04)	1.24 (0.04)
Number of friends^a^	Mean (SE)	5.10 (0.08)	5.11 (0.08)
Perceived social support^a^	Socially isolated	669 (16.1)	16.1
	Not socially isolated	3,485 (83.9)	83.9
**EXPOSURE**			
Childhood adversity^a^^,^ ^d^	Mean (SE)	2.91 (0.02)	2.91 (0.02)
	0	78 (2.1)	2.0
	1	311 (8.4)	8.0
	2	794 (21.3)	21.5
	3	1,462 (39.3)	39.5
	4	897 (24.1)	24.2
	5	133 (3.6)	3.4
	6	46 (1.2)	1.2
	7	4 (0.1)	0.1
**MEDIATOR**			
Daily smoking^a^	Yes	1,312 (29.0)	29.0
	No	3,213 (71.0)	71.0
**RESPONSE**			
Anxious and depressive symptomatology (HSCL-10 score)^a^^,^ ^e^	Mean (SE)	1.22 (0.01)	1.25 (0.01)

a *The numbers for some variables do not add up to 4,530 due to missing values*.

b *There were no missing values, so no imputations were made for these variables*.

c *Education is measured on a 5-level scale: (1) college or university (4 years or more); (2) college or university (less than 4 years); (3) high school diploma; (4) vocational school or technical school; and (5) primary and secondary school or similar (i.e., 7–10 years of schooling)*.

d *The seven childhood adversities considered were: low mother's and father's education (parental primary and secondary school or similar), difficult or very difficult subjective childhood financial conditions. psychological abuse, physical abuse, substance abuse distress, and exposure to passive smoke in childhood*.

e *HSCL-10 (1.0–4.0), where 1.0 represents lowest score on anxious and depressive symptomatology, and 4.0 represents highest score on anxious and depressive symptomatology*.

*SE, standard error; HSCL-10, Hopkins Symptom Check List-10*.

The association between childhood adversity and smoking in adulthood was assessed by Poisson regression analysis with a robust error variance (Zou, 2004). The association between smoking in adulthood and ADS in adulthood was assessed by ordinary least square (OLS) regression analysis. Relative risks (RRs), OLS estimates (β) and corresponding 95% CIs are presented. A nonlinear association between childhood adversity and ADS in adulthood was observed. The cubic form was identified as the best fitting fractional polynomial; therefore, the cubic form was used in all analysis, to account for the nonlinear association. Childhood adversity was transformed as:

$$\text{Childhood }{\text{adversity}}_{\text{transformed}}=(1+\text{Childhood }{\text{adversity}}^{3})/100$$

### Assessing direct and indirect effects (via smoking) of childhood adversity on anxious and depressive symptomatology in adulthood

Mediation analysis with the difference-in-coefficients method (Shrout and Bolger, 2002; MacKinnon et al., 2007; Sheikh et al., 2016b) was used. No statistically significant multiplicative interaction between childhood adversity and smoking (*p* > 0.1) was observed in this sample. Four estimates are presented: total effects (adjusted for confounding variables), direct effects (adjusted for confounding variables and smoking), indirect effects (difference between total effect and direct effect), and proportion mediated (%). Smoking in adulthood was included in the models to assess the indirect effect and the proportion of mediated effect (%). If smoking in adulthood is an important mediator of the association between childhood adversity and ADS in adulthood, the effects of childhood adversity (β_Total Effect_) should decline when smoking in adulthood is added to the model. Since education may be an intermediate confounder in the CA → smoking → ADS association, we assessed whether education was a mediator between CA and smoking. However, the indirect effect of CA on smoking (via education) was not statistically significant. Therefore, we ruled out the possibility that education is an intermediate confounder in this study sample (data not shown).

SEs were derived with bootstrapping for hypothesis testing, and 95% CIs are presented. Furthermore, in order to assess whether the proportion of mediated effects varies due to the smoking-ADS confounding factors included in the model, each smoking-ADS confounding factor was omitted from the models, one-by-one.

## Results

The majority of the participants in our study sample were aged 55 and over (56.2%) in Tromsø IV and were female (59.2%) (Table 1). The average number of childhood adversities in this sample was 2.91 (SE: 0.02) (Table 1). Only 2.1% of the participants reported none of the childhood adversities considered in this study, while 8.4% of the participants had experienced at least one (Table 1). Almost one-third (29.0%) of the participants were daily smokers, and the mean score on ADS was 1.25 (SE: 0.01) in Tromsø V (Table 1).

In the fully-adjusted model, childhood adversity was associated with a 10% higher risk of being a daily smoker in adulthood (RR: 1.10, 95% CI: 1.03; 1.18) (Table 2). Moreover, in the fully-adjusted model, daily smoking in adulthood was associated with greater levels of ADS in adulthood (β: 0.07, *p* < 0.001) (Table 3).

**Table 2:** Association between childhood adversity and smoking in adulthood (*n* = 4,530).

	**Smoking in adulthood**			
	**Crude**		**Adjusted^c^**	
	**RR**	**95% CI**	**RR**	**95% CI**
Childhood adversity	1.09^a^	1.01, 1.17	1.10^b^	1.03, 1.18

a *p < 0.01*.

b *p < 0.001*.

c *Adjusted for age, gender, living in Norway at age 1 year, mother's/father's history of psychological problems, parental history of heart attack/angina pectoris cerebral stroke/brain hemorrhage/osteoporosis/stomach/duodenal ulcer asthma/diabetes/dementia*.

*RR, relative risk; CI, confidence interval*.

**Table 3:** Association between smoking and anxious and depressive symptomatology (*n* = 4,530).

	**Anxious and depressive symptomatology**			
	**Crude**		**Adjusted^b^**	
	**β**	**95% CI**	**β**	**95% CI**
Smoking	0.07^a^	0.05, 0.10	0.07^a^	0.04, 0.10

a *p < 0.001*.

b *Adjusted for age, gender, living in Norway at age 1 year, parental education, subjective childhood financial conditions, psychological abuse in childhood, physical abuse in childhood, substance abuse distress in childhood, exposure to passive smoke in childhood, mother's/father's history of psychological problems, parental history of heart attack/angina pectoris/cerebral stroke/brain hemorrhage/osteoporosis/stomach/duodenal ulcer/asthma/diabetes/dementia, alcohol intake indicators, education, number of friends, and perceived social support*.

*CI, Confidence interval*.

Table 4 presents the total effect, direct effect, indirect effect (via smoking in adulthood), and proportion of mediated effect (via smoking in adulthood) of childhood adversity on ADS in adulthood. After controlling for all the exposure-mediator, mediator-response, and exposure-response confounding variables considered in this study, childhood adversity was associated with greater levels of ADS in adulthood (β_Total Effect_: 0.064, 95% CI: 0.045; 0.083). Decomposition of total effects showed that almost all of the effect of childhood adversity was independent of smoking in adulthood (β_Direct Effect_: 0.063, 95% CI: 0.043; 0.078). Consequently, the indirect effect (β_Indirect Effect_: 0.001, *p* > 0.05) and proportion of mediated effect (%_Proportion mediated_: 1.30, *p* > 0.05) were not statistically significant (Table 4).

**Table 4:** Direct and indirect effect (via smoking) of childhood adversity on anxious and depressive symptomatology in adulthood (*n* = 4,530).

	**Adjusted for all Confounding Variables**			
	**Total effect β (95% CI)**	**Direct effect β (95% CI)**	**Indirect effect β (95% CI)**	**Proportion mediated % (95% CI)**
Childhood adversity	0.064 (0.045, 0.083)^a^	0.063 (0.043, 0.078)^b^	0.001 (−0.001, 0.002)^a^	1.30 (−1.05, 3.81)^a^
	**Excluding Education**			
	0.069 (0.040, 0.085)^e^	0.067 (0.039, 0.079)^f^	0.002 (0.001, 0.004)^e^	2.74 (0.06, 5.37)^e^
	**Excluding Indicators of Alcohol Intake**			
	0.064 (0.044, 0.081)^c^	0.063 (0.043, 0.079)^d^	0.001 (−0.001, 0.002)^c^	1.50 (−0.50, 4.20)^c^
	**Excluding Indicators of Social Support**			
	0.074 (0.055, 0.092)^g^	0.074 (0.054, 0.089)^h^	0.001 (−0.001, 0.002)^g^	1.05 (−0.58, 3.39)^g^

a *Adjusted for age, gender, living in Norway at age 1 year, mother's/father's history of psychological problems, parental history of heart attack/angina pectoris/cerebral stroke/brain hemorrhage/osteoporosis/stomach/duodenal ulcer/ asthma/diabetes/dementia, education, number of friends, perceived social support, and indicators of alcohol intake*.

b *Adjusted for age, gender, living in Norway at age 1 year, mother's/father's history of psychological problems, parental history of heart attack/angina pectoris/cerebral stroke/brain hemorrhage/osteoporosis/stomach/duodenal ulcer/asthma/diabetes/dementia, education, number of friends, perceived social support, indicators of alcohol intake + smoking in adulthood*.

c *Adjusted for age, gender, living in Norway at age 1 year, mother's/father's history of psychological problems, parental history of heart attack/angina pectoris/cerebral stroke/brain hemorrhage/osteoporosis/stomach/duodenal ulcer/asthma/diabetes/dementia, education, number of friends, and perceived social support*.

d *Adjusted for age, gender, living in Norway at age 1 year, mother's/father's history of psychological problems, parental history of heart attack/angina pectoris/cerebral stroke/brain hemorrhage/osteoporosis/stomach/duodenal ulcer/asthma/diabetes/dementia, education, number of friends, and perceived social support + smoking in adulthood*.

e *Adjusted for age, gender, living in Norway at age 1 year, mother's/father's history of psychological problems, parental history of heart attack/angina pectoris/cerebral stroke/brain hemorrhage/osteoporosis/stomach/duodenal ulcer/asthma/diabetes/dementia, number of friends, perceived social support, and indicators of alcohol intake*.

f *Adjusted for age, gender, living in Norway at age 1 year, mother's/father's history of psychological problems, parental history of heart attack/angina pectoris/cerebral stroke/brain hemorrhage/osteoporosis/stomach/duodenal ulcer/asthma/diabetes/dementia, number of friends, perceived social support, indicators of alcohol intake + smoking in adulthood*.

g *Adjusted for age, gender, living in Norway at age 1 year, mother's/father's history of psychological problems, parental history of heart attack/angina pectoris/cerebral stroke/brain hemorrhage/osteoporosis/ stomach/duodenal ulcer/asthma/diabetes/dementia, education, number of friends, perceived social support, and indicators of alcohol intake*.

h *Adjusted for age, gender, living in Norway at age 1 year, mother's/father's history of psychological problems, parental history of heart attack/angina pectoris/cerebral stroke/brain hemorrhage/osteoporosis/stomach/duodenal ulcer/asthma/diabetes/dementia, education, number of friends, perceived social support, indicators of alcohol intake + smoking in adulthood*.

CI, Confidence interval

Smoking-ADS confounding factors (i.e., education, alcohol intake, and social support) were excluded from the models one-by-one, in order to assess how the indirect effect and proportion of mediated effect changed (Table 4). Excluding indicators of alcohol intake or social support did not change the inference, as the indirect effects and proportion of mediated effects were not statistically significant (*p* > 0.05). However, when education was excluded from the models, smoking in adulthood significantly (%_Proportion mediated_: 2.74, *p* < 0.05) mediated the association between childhood adversity and ADS in adulthood (Table 4).

## Discussion

The results of this study show that childhood adversity is associated with an increased risk of smoking in adulthood, and smoking in adulthood is associated with greater levels of ADS in adulthood. The direct effects presented in this study show that the effects of childhood adversity on ADS in adulthood were slightly attenuated, but not eliminated, by adjustment for smoking in adulthood. Moreover, the results of this study show that a careful inclusion of mediator-response confounding variables is not only important for a “less” biased estimates of direct effect, indirect effect, and proportion of mediated effect, but also for the binary decision of whether there is an indirect effect based only on statistical significance. Indeed, if education was not measured or not considered as a potential confounder, a researcher relying on significance tests would conclude that smoking in adulthood significantly (*p* < 0.05, Table 4) mediates the association between childhood adversity and ADS in adulthood.

Conditional on all the confounding variables considered in this study, smoking in adulthood did not significantly mediate the association between childhood adversity and ADS in adulthood. However, if excluding one mediator-response confounding variable (education) can make the indirect effect appear statistically significant, it is possible that including other confounding variables could also influence statistical significance. Similarly, it must be noted that despite accounting for several confounding variables and establishing temporality between smoking and ADS in adulthood, the indirect effects presented here cannot be interpreted as causal effects because there may be unmeasured (or unaccounted for) exposure-mediator, mediator-response, or exposure-response confounding variables, as well as unmeasured intermediate confounding variables (Sheikh et al., 2016b; Sheikh, 2017). Although childhood adversity was the focus of the study, we cannot rule out that exposure to adverse events later in life might have affected smoking and ADS. Therefore, the results presented here do not “prove” that the indirect effect of childhood adversity on ADS via smoking is not causal, as the assumption of no unmeasured confounding is not satisfied, and may never be satisfied using an observational study design (Sheikh et al., 2016b; Sheikh, 2017). One might wonder what the purpose of such assumptions is if they are unlikely to be satisfied. We believe that the purpose, in practice, is to urge researchers to provide “as unbiased as feasible” estimates, instead of “as biased as possible” estimates. Unbiased estimates with a causal interpretation from observational studies represent an unattainable goal, but at least researchers can aim to reduce the “error” in estimates.

It must be noted that the inference regarding indirect effects is often based on statistical significance. This approach has been criticized, but for most journals publication of a manuscript relies (directly or indirectly) on statistical significance. This is why we chose to highlight the statistical significance in the contradictory inference drawn from indirect effects/proportion mediated effects when education is included or excluded from the models. If statistical significance is ignored, than all the indirect effects and proportion mediated effects in Table 4 provide the same information, i.e., some of the effect of childhood adversity on ADS in adulthood is mediated by smoking in adulthood, as the indirect effects were >0, and proportion mediated effects in all models were:

0<Proportion Mediated(%)≤100.

Consistent with previous studies, childhood adversity was associated with smoking in adulthood (Rohde et al., 2004; Le et al., 2016; Non et al., 2016) and with ADS in adulthood (Mauramo et al., 2012; Nurius et al., 2012; Quesnel-Vallée and Taylor, 2012; Baldassari et al., 2013; Kamiya et al., 2013; Oshio et al., 2013; Sperry and Widom, 2013; Torres and Wong, 2013; Ochi et al., 2014; Sheikh et al., 2014; Taha and Goodwin, 2014). Similarly, and consistent with most (Jorm et al., 1999; Johnson et al., 2000; Martini et al., 2002; Nicolosi et al., 2004; Boden et al., 2010; Sheikh et al., 2014, 2016a), though not all (Kendler et al., 1993b; Albers and Biener, 2002; Nabi et al., 2010) previous studies, smoking in adulthood was associated with ADS in adulthood. Several previous studies (Kendler et al., 1993b; Albers and Biener, 2002; Fergusson et al., 2003) have suggested that including potential confounding variables in the models substantially reduces the strength of association between smoking and ADS. However, the strength of association between smoking and ADS remained similar in this study, despite adjusting for a wide range of confounding variables. Contrary to some studies (Sheikh et al., 2014), though not all (Gilman et al., 2002; Park et al., 2013; Sheikh et al., 2016a; Dahl et al., 2017; Sheikh, 2017), no statistically significant interaction was observed between childhood adversity and gender. Similarly, contrary to some previous studies (Lin et al., 2014), no statistically significant interaction between smoking and gender was observed in this sample. Furthermore, age (cohort effect Etherington, 2017) did not play a moderating role in the associations between childhood adversity, smoking, and ADS in adulthood (data not shown), which is in contrast to some (Goosby, 2013; Schaan, 2014), though not all (Raposo et al., 2014; Sheikh et al., 2014, 2016a,b; Sheikh, 2017) previous studies.

Statistically, the change in the estimate for an exposure due to a confounder or a mediator may be assessed in the same manner (with either the difference method or with statistical methods based on counterfactuals/potential outcomes) (Sheikh et al., 2016b). Therefore, the estimates for direct effects can be interpreted as an alternative hypothesis of whether childhood adversity affects ADS in adulthood, independent of smoking. This interpretation is sometimes preferred (instead of mediation hypothesis) for two reasons: first, smoking tends to be affected by parental behavioral patterns irrespective of psychosocial and socioeconomic conditions in childhood (Broms et al., 2012). Therefore, some of the association between childhood adversity and ADS may be driven by the association between parental behavioral patterns (Taha and Goodwin, 2014), and participants' smoking behavior. Second, the association between smoking and ADS may be reciprocal. For instance, smoking may be used to alleviate feelings of depression due to serotonergic dysfunctions (Stahl, 2013). Childhood adversity could negatively influence anxiety and depression in adolescence (Raposa et al., 2014; Wirback et al., 2014; Heshmat et al., 2016; Seijo et al., 2016; Björkenstam et al., 2017; Kang et al., 2017), which in turn may influence smoking behavior in adulthood. Indeed, history of anxiety and depression is also a strong predictor of anxiety and depression in later-life (Guerra et al., 2009; Din and Noor, 2010; Chen et al., 2012; Björkenstam et al., 2015; Davies et al., 2015).

The test-retest reliability of some variables was not very good in this study sample. The unreliability of confounding variables would imply that the total effects, direct effects, indirect effects and proportion of mediated effects are over-estimated (biased upwards) (Sheikh et al., 2016b). However, the unreliability of mediator (smoking) would imply that the indirect effects and proportion of mediated effects are under-estimated (biased downwards) (Sheikh et al., 2016b). Several studies have shown that retrospective measurement of childhood adversity is fairly reliable and valid (Krieger et al., 1998; Ward, 2011; Goodman et al., 2016). Other studies have shown that the association between retrospectively measured childhood adversity and ADS in adulthood is not driven by differential recall bias (Brown et al., 2007; Fergusson et al., 2011; Sheikh, 2017). The adverse effects of smoking are in part a function of the number of cigarettes smoked and duration of smoking. As this analysis did not have data on the amount or duration of smoking, the indirect effects could be biased downwards (under-estimated). The follow-up period between smoking and ADS was only 7 years; therefore, it is plausible that smoking may mediate the childhood adversity-ADS association over a longer period of time. No other psychometric scales were measured in the Tromsø Study and ADS was not measured in the Tromsø IV Study. Including ADS from an earlier wave (Tromsø IV) could strengthen the argument that smoking contributes a unique variance to subsequent ADS in Tromsø V. The strengths of this study are its longitudinal design and a large representative sample of the adult population of Tromsø.

In summary, the results of this study suggest that childhood adversity is associated with greater levels of anxiety and depression in adulthood, and this association was not mediated significantly by smoking in adulthood.

## Author contributions

The author confirms being the sole contributor of this work and approved it for publication.

### Conflict of interest statement

The author declares that the research was conducted in the absence of any commercial or financial relationships that could be construed as a potential conflict of interest.
